# Stoneflies of the genus *Neoperla* (Plecoptera, Perlidae) from Wuyi Mountain National Nature Reserve, Fujian of China

**DOI:** 10.3897/zookeys.326.5911

**Published:** 2013-08-22

**Authors:** Xue-Feng Qin, Dávid Murányi, Guo-Quan Wang, Wei-Hai Li

**Affiliations:** 1 Department of Plant Protection, Henan Institute of Science and Technology, Xinxiang, Henan 453003, China; 2 Department of Zoology, Hungarian Natural History Museum, Baross u. 13, H-1088 Budapest, Hungary; 3 Department of Plant Protection, Guangxi University, Nanning, Guangxi 530004, China

**Keywords:** Plecoptera, Perlidae, *Neoperla*, new species, China

## Abstract

The species of the genus *Neoperla* are reviewed from Wuyi Mountain National Nature Reserve located in the Fujian Province of southeastern China, including the description of a new species, *Neoperla brevistyla* **sp. n.** The new species is compared to similar taxa. The first records of five *Neoperla* species, *Neoperla henana* Li, Wu & Zhang, 2011, *Neoperla similiserecta* Wang & Li, 2012, *Neoperla qingyuanensis* Yang & Yang, 1995, *Neoperla xuansongae* Li & Li, 2013 and *Neoperla tuberculata* Wu, 1938 are given for the Wuyi Mountain. A provisional key is provided for facilitating the identification of these species.

## Introduction

The stonefly genus *Neoperla* is the most species-rich genus within the subfamily Perlinae (Sivec et al. 1988, DeWalt et al. 2013). There are over 70 known species in China described by Chu (1929), Du (1999, 2000a, 2000b), Du and Sivec (2004, 2005), Du and Wang (2005, 2007), Du et al. (1999, 2001), Sivec and Zwick (1987), Wu (1935, 1938, 1948, 1962, 1973), Wu and Claassen (1934), Yang and Yang (1990, 1991), Yang and Yang (1992, 1993, 1995a, 1995b, 1996, 1998), Li et al. (2011), Li et al. (2011), Li and Wang (2011), Li et al. (2012), Li et al. (2012), Li and Li (2013), Li et al. (2013a) and Li et al. (2013b).

In the present paper, the species ofthe genus *Neoperla* are reviewed from Wuyi Mountain National Nature Reserve, Fujian Province of China based on the fresh material collected in recent years. With a total area of ca. 565 km^2^, the Reserve possesses a typical subtropical forest ecological system and is the largest area for biodiversity conservation in Fujian Province, south-east China (http://www.wbr.cn/).

## Material and methods

Types and other examined material are deposited in the Insect Collection of Henan Institute of Science and Technology (HIST), Xinxiang, and the Entomological Museum of China Agricultural University (CAU), Beijing. They were examined with the aid of a Motic SMZ 168 microscope and the color illustrations were captured using digitalized software Motic Images Advanced 3.2. All specimens were kept in 75% ethanol. Aedeagi were everted using the cold maceration technique of Zwick (1983). Terminology follows that of Sivec et al. (1988).

## Taxonomy

### Neoperla breviscrotata

Du, 1999

http://species-id.net/wiki/Neoperla_breviscrotata

Neoperla breviscrotata Du, 1999: 312. Type locality: Guizhou Province, Sandou, Chengguan.

#### Distribution.

China (Guizhou, Anhui, Shandong, Fujian, Shaanxi).

#### Remarks.

This species was recently described by Du (1999) from the localities listed above including the Wuyi Mountain, but no new material was available to the present study.

### Neoperla brevistyla

Li & Murányi sp. n.

http://zoobank.org/6FB22544-4438-4E18-8E5C-4F68C9BB1498

http://species-id.net/wiki/Neoperla_brevistyla

Figs 1 –2

#### Type material.

1 male (CAU), China: Fujian Province, Mt. Wuyishan, Sangang, 735 m, 27°74.78'N, 117°68.31'E, light trap, 12 Jul. 2009, Li Shi and Xiaoyan Liu. Paratypes: 2 males, the same data as holotype, (CAU); 6 males, the same locality, 16 Aug. 2006, Hui Dong, (HIST).

#### Male.

**Figure 1.**
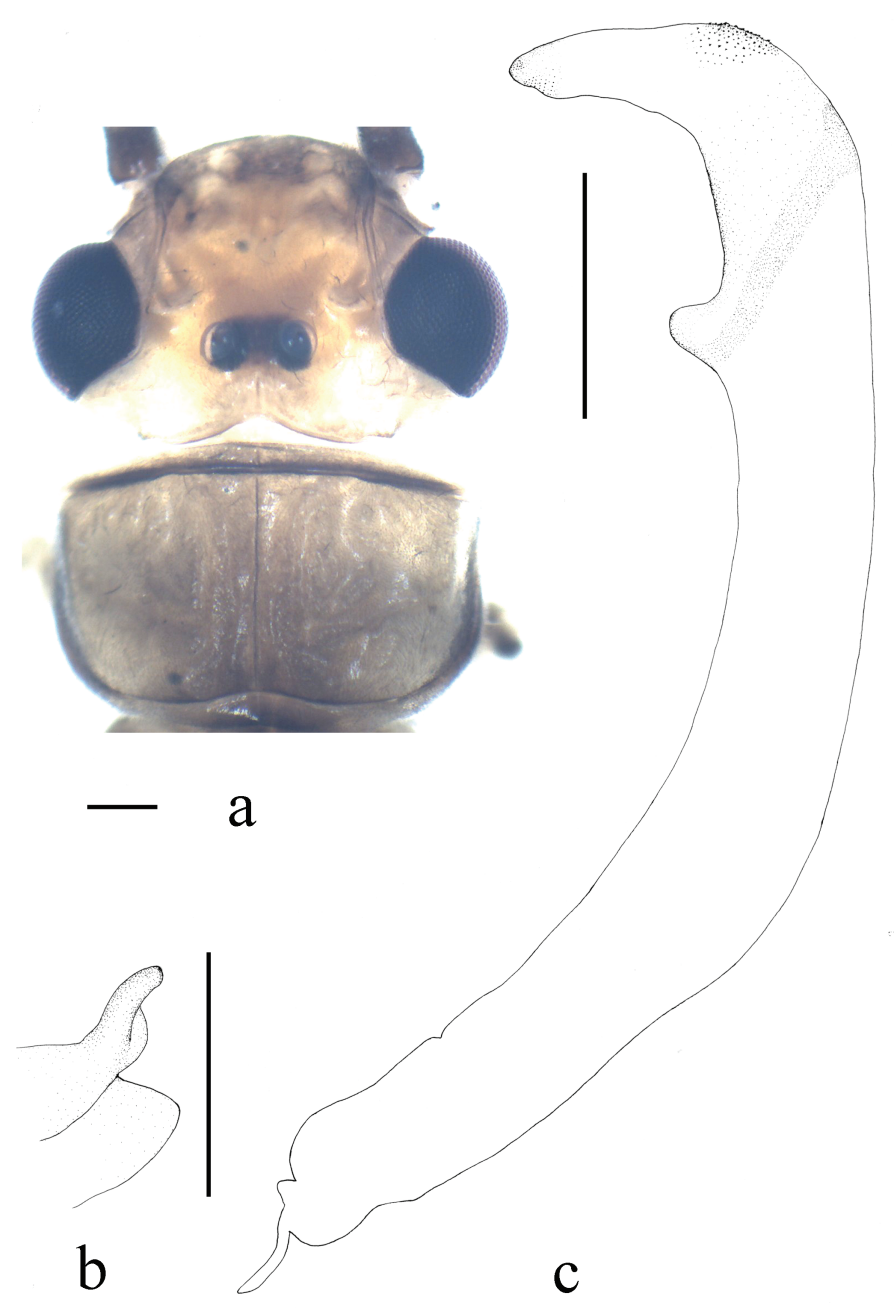
*Neoperla brevistyla* Li & Murányi, sp. n. Male **a** Head and pronotum, dorsal view **b** Hemitergal process, dorsal view **c** Aedeagus, lateral view. Scale bars: 0.5 mm.

**Figure 2.**
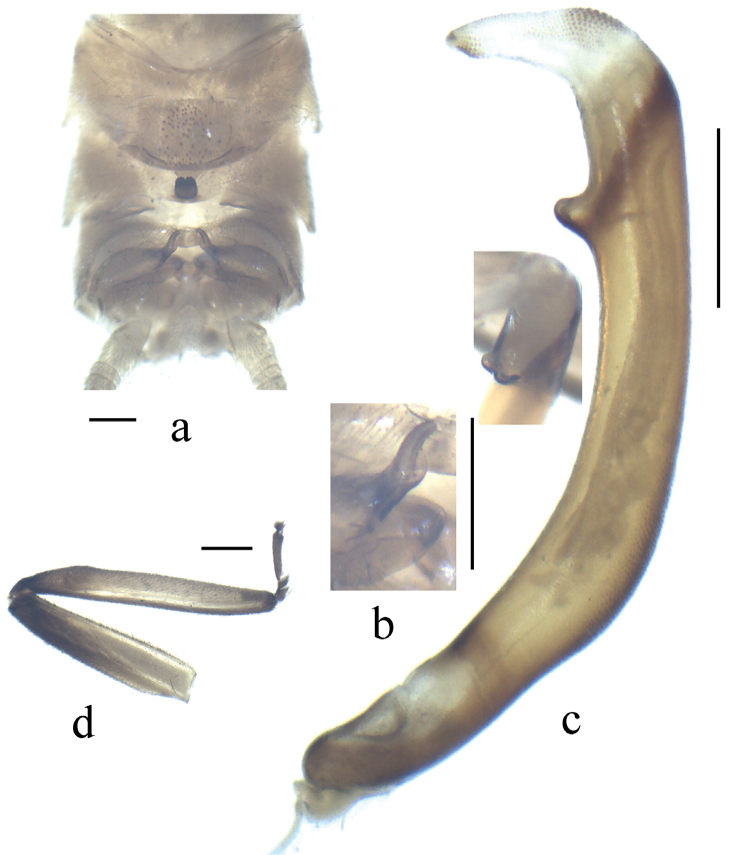
*Neoperla brevistyla* Li & Murányi,sp. n. Male **a** Terminalia, dorsal view **b** Hemitergal process, dorsal view **c** Aedeagus, lateral view **d** Foreleg, lateral view. Scale bars: 0.5 mm.

Forewing length 15.6–16.0 mm. Distance between ocelli about as wide as diameter of the ocellus. Head slightly wider than pronotum, with an obscure quadrate dark stigma on frons and a brown area covering ocelli (Fig. 1a); compound eyes black and antennae dark; maxillary palpi brown. Pronotum with obscure rugosities and dark brown anterior and lateral margins, thorax mostly brown (Fig. 1a); wing membrane subhyaline, veins brown; legs brown but distal half of femora and basal one fourth and distal one sixth of tibia dark brown, tarsi dark brown (Fig. 2d).

*Terminalia*. The posterior margin of tergum 7 with quadrate, rounded and elevated process covered with dense sensilla basiconica (Fig. 2a). Tergum 8 with recurved tongue-like process (Figs 2a). Tergum 9 without sensilla basiconica. Hemitergal processes of tergum 10 with enlarged base and beak-like apex (Figs 1b and 2b). Aedeagal tube strongly sclerotized, with a pair of separate ventroapical lobes, in lateral view nipple like and triangular in shape (Figs 1c and 2c). Aedeagal sac short and gradually tapering to apex, forming nearly a right angle with tube; basal part unarmed and plump, median part with a patch of small spines and apex bearing fine spinules (Figs 1c and 2c).

#### Female.

Unknown.

#### Etymology.

The specific epithet refers to the short ventral lobe of aedeagal tube.

#### Distribution.

China (Fujian).

#### Diagnosis and remarks.

**Figure 3.**
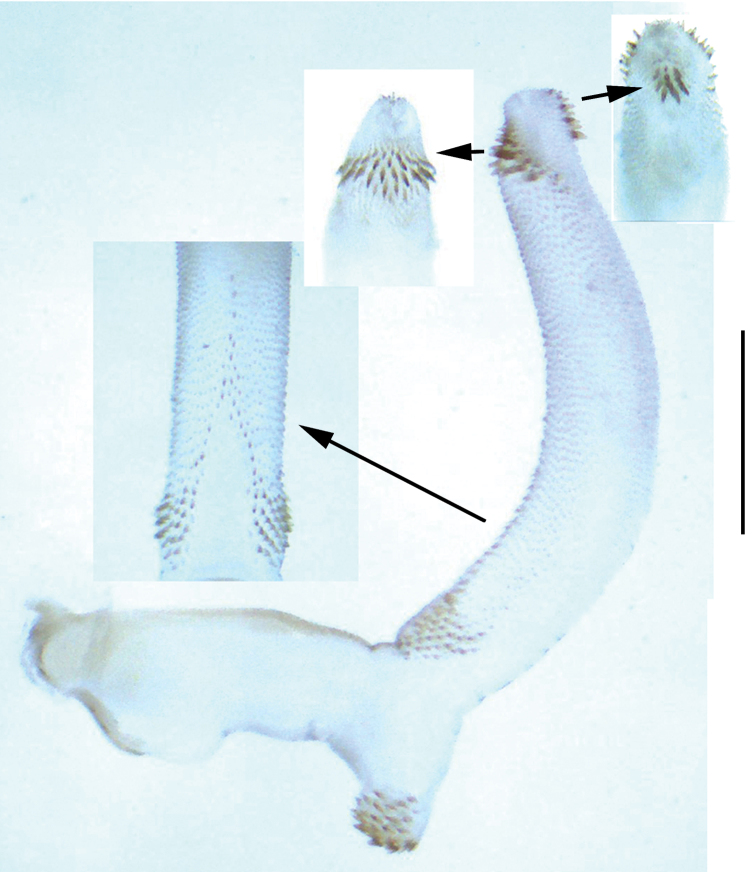
*Neoperla henana*. Male aedeagus, lateral view. Scale bar: 0.5 mm.

**Figure 4.**
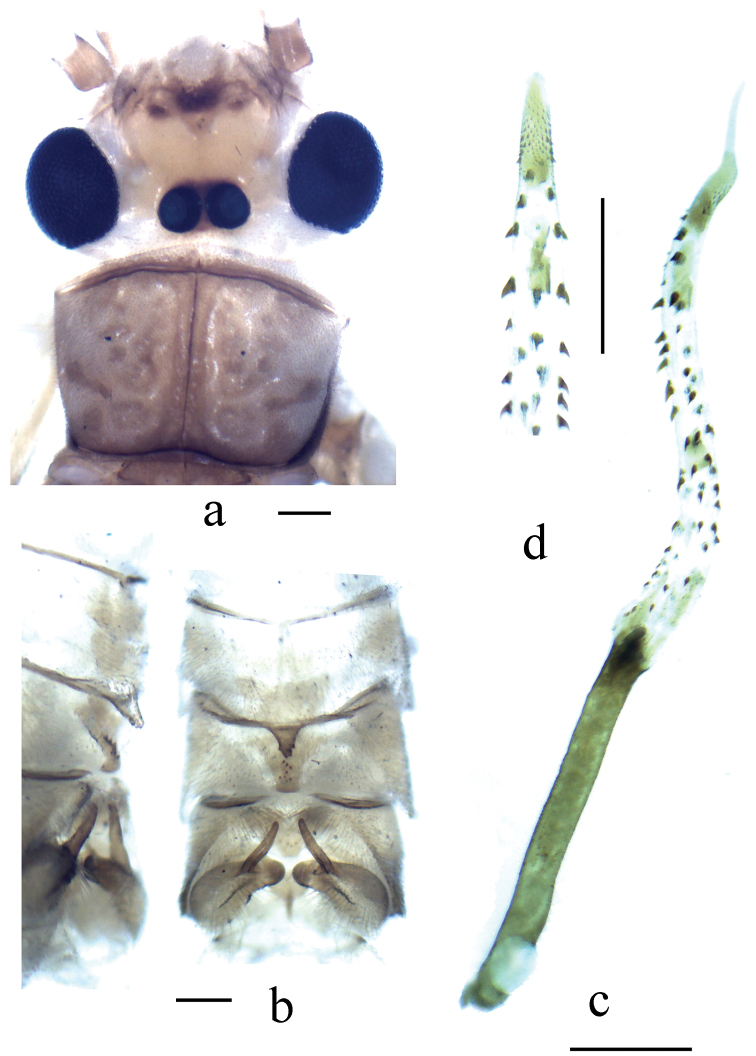
*Neoperla lii*. Male **a** Head and pronotum, dorsal view **b** Terminalia, dorsal view **c** Aedeagus, lateral view **d** Distal half of aedeagal sac, ventral view. Scale bars: 0.5 mm.

*Neoperla brevistyla* belongs to an informal group of species including *Neoperla biprojecta* Du, 2001, *Neoperla duratubulata* Du, 1999, *Neoperla qingyuanensis* Yang & Yang, 1995, *Neoperla yentu* Cao & Bae, 2007 that bear similar terminalia, sclerotized aedeagal tube and short sac, and a pair of separate ventral lobes of aedeagal tube (for comparison, see figs 3–4, 7 in Li et al. 2013, figs 25–13 in Du 1999, figs 75–77 in Stark and Sivec 2008, and Fig. 6). This species shares a pair of short ventral aedeagal lobes with *Neoperla biprojecta* Du, 2001 (fig. 4 in Li et al. 2013), but the lobes of *Neoperla brevistyla* are triangular andstouter, a patch of dorsal armatures occurs at midlength of the sac (Fig. 1c) which is absent in *Neoperla biprojecta*.

### Neoperla duratubulata

Du, 1999

http://species-id.net/wiki/Neoperla_duratubulata

Neoperla duratubulata Du, 1999: 313. Type locality: Fujian Province, Mt. Wuyishan, Sangang.

#### Remarks.

No new material was available for examination. It was stated to be similar to *Neoperla flavescens* Chu, 1929 but bearing paired ventral aedeagal lobes (Du 1999). *Neoperla duratubulata* may be assigned to the above mentioned subgroup and seems similar to *Neoperla qingyuanensis*. The aedeagal sac of *Neoperla duratubulata* is a very short conical structure with several rows of subapical fine spinules and the head lacks distinct color pattern (modified from the original descriptions of Du 1999).

### Neoperla henana

Li, Wu & Zhang, 2011

http://species-id.net/wiki/Neoperla_henana

Fig. 3

Neoperla henana Li, Wu & Zhang, 2011: 33. Type locality: Henan Province, Nanyang, Laojieling.

#### Material examined.

2 males, (CAU), China: Fujian Province, Mt. Wuyishan, Sangang, 27°74.78'N, 117°68.31'E, light trap, 6 Aug. 2006, Hui Dong.

#### Distribution.

China (Fujian and Henan provinces).

#### Remarks.

This species was recently described by Li et al. (2011). The color pattern and terminalia among the types and present material show no variations. The type material (figs 1-3 in Li et al. 2011) seems two teneral specimens where the fine spinules of aedeagal sac are not as well developed as of the present ones (Fig. 3).

### Neoperla lii

Du, 1999

http://species-id.net/wiki/Neoperla_lii

Figs 4 –5

Neoperla lii Du, 1999: 315. Type locality: Fujian Province, Mt. Wuyishan, Sangang.

#### Material examined.

1 male, (CAU), China: Fujian Province, Mt. Wuyishan, Sangang, 27°74.78'N, 117°68.31'E, light trap, 19 Aug. 2006, Xian Zhou; 4 males, same locality, 25 Sep. 2009, Tingting Zhang, (HIST).

#### Distribution.

China (Fujian).

#### Remarks.

This species was recently described by Du (1999). The original illustrations are rather small in print and show no details on the fine armatures of the sac but the detailed descriptions agree well with the fresh material. We present herein the detailed illustrations of the aedeagal sac for a better diagnosis of *Neoperla lii*.

**Figure 5.**
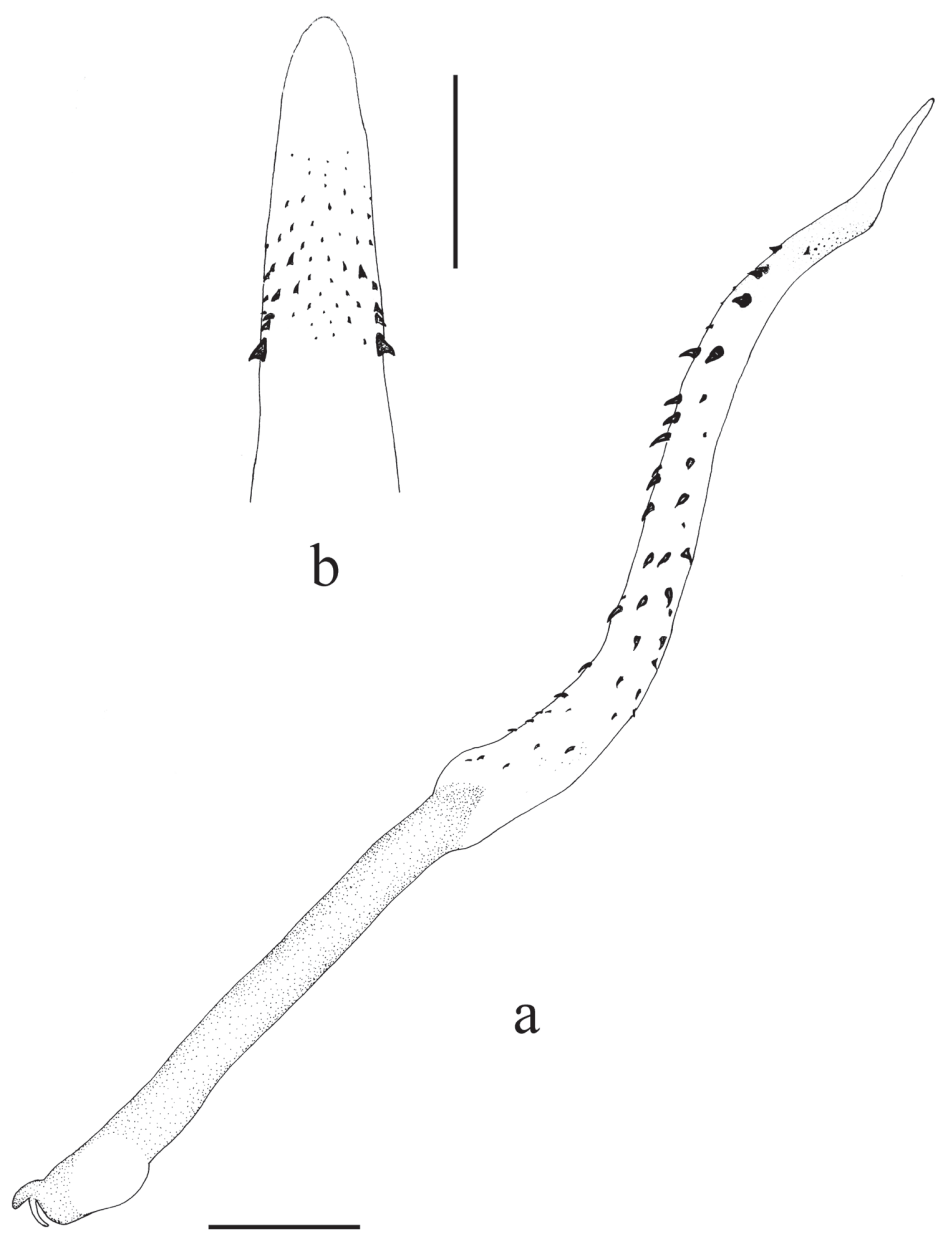
*Neoperla lii*. Male **a** Aedeagus, lateral view **b** Apex of aedeagal sac, ventral view. Scale bars: 0.5 mm.

### Neoperla qingyuanensis

Yang & Yang, 1995

http://species-id.net/wiki/Neoperla_qingyuanensis

Fig. 6

Neoperla qinyuanensis Yang & Yang, 1995b: 59; Li et al. 2013: 361. Type locality: Zhejiang Province, Qingyuan County, Mt. Baishanzu, Chameiyu.

#### Material examined.

2 males, (CAU), China: Fujian Province, Mt. Wuyishan, Sangang, 27°74.78'N, 117°68.31'E, light trap, 6 Aug. 2006, Hui Dong; 1 male, same locality, 19 Aug. 2006. Xian Zhou.

#### Distribution.

China (Zhejiang, Fujian).

#### Remarks.

This species was recently redescribed by Li et al. (2013). The head patterns of the examined types were faded. The illustrations of head pattern and fully extruded aedeagus are provided here on the fresh material to facilitate the identifying of *Neoperla qinyuanensis*.

**Figure 6.**
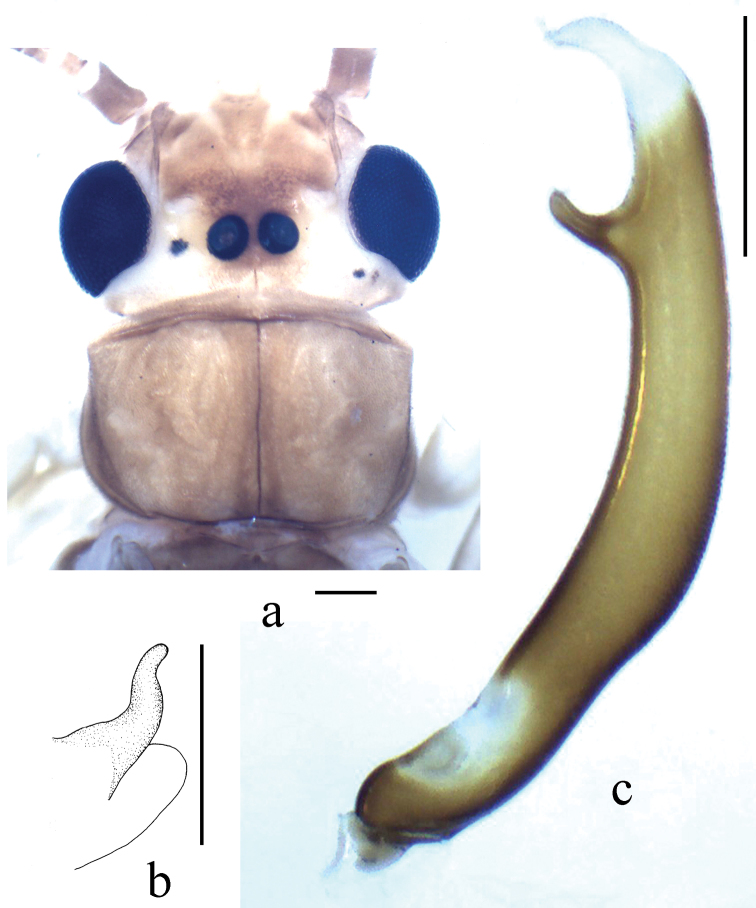
*Neoperla qingyuanensis*. Male **a** Head and pronotum, dorsal view **b** Hemitergal process, dorsal view **c** Aedeagus, lateral view. Scale bars: 0.5 mm.

### Neoperla tuberculata

Wu, 1938

http://species-id.net/wiki/Neoperla_tuberculata

Fig. 7

Neoperla tuberculata Wu, 1938: 122; Du et al. 2001: 77. Type locality: Zhejiang Province, Mt. Tianmushan.

#### Material examined.

4 males, (CAU), China: Fujian Province, Mt. Wuyishan, Sangang, 27°74.78'N, 117°68.31'E, 9 May 2004, Xingyue Liu.

#### Remarks.

**Figure 7.**
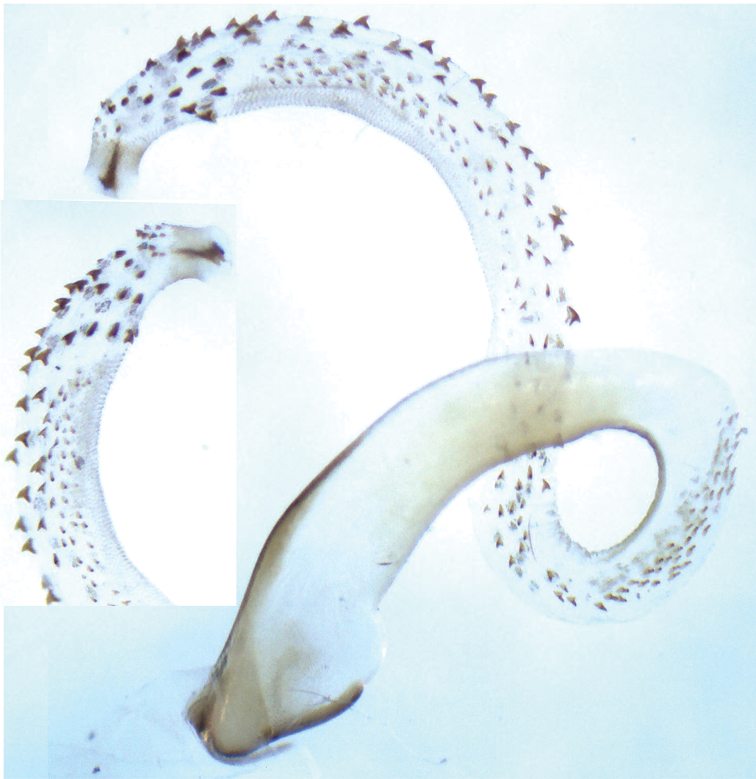
*Neoperla tuberculata*. Male aedeagus, lateral view. Scale bars: 0.5 mm.

Our specimens bear slight variation in the aedeagal sac armatures (Fig. 7): the subapical cluster of larger spines in Zhejiang specimens are not prominent in these ones; but they agree well with the Zhejiang one in terminalia features, the head and leg patterns.

### Neoperla similidella

Li & Wang, 2013

http://species-id.net/wiki/Neoperla_similidella\according to Qin et al 2013

Neoperla similidella Li & Wang, 2013: 25. Type locality: China: Fujian Province, Mt. Wuyishan, Kekao Station.

#### Additional material examined.

3 males, (CAU), China: Fujian Province, Mt. Wuyishan, Sangang, 27°74.78'N, 117°68.31'E, light trap, 27 Jun. 2009, Li Shi and Xiaoyan Liu.

#### Distribution.

China (Fujian Province).

#### Remarks.

This species was recently recognized by Li et al. (2013) from Wuyi Mountain.

### Neoperla similiserecta

Wang & Li, 2012

http://species-id.net/wiki/Neoperla_similiserecta

Neoperla similiserecta Wang & Li, 2012: 18. Type locality: China: Guangdong Province, Xinfeng County, Mt. Yunjishan.

#### Material examined.

2 males, (CAU), China: Fujian Province, Mt. Wuyishan, Sangang, 27°74.78'N, 117°68.31'E, light trap, 12 Jul. 2009, Shi Li and Liu Xiao-Yan.

#### Distribution.

China (Fujian and Guangdong provinces).

#### Remarks.

This species was recently described from Guangdong by Li et al. (2012). The present specimens agree well with the type material.

### Neoperla xuansongae

Li & Li, 2013

http://species-id.net/wiki/Neoperla_xuansongae

Fig. 8

Neoperla xuansongae Li & Li, 2013: 362. Type locality: Zhejiang Province, Li’an County, Mt. Tianmushan, Sanmuping.

#### Material examined.

4 males, (CAU), China: Fujian Province, Mt. Wuyishan, Sangang, 27.7478'N, 117.6831'E, 16 Aug. 2006, Hui Dong.

#### Distribution.

China (Fujian and Zhejiang provinces).

#### Remarks.

**Figure 8.**
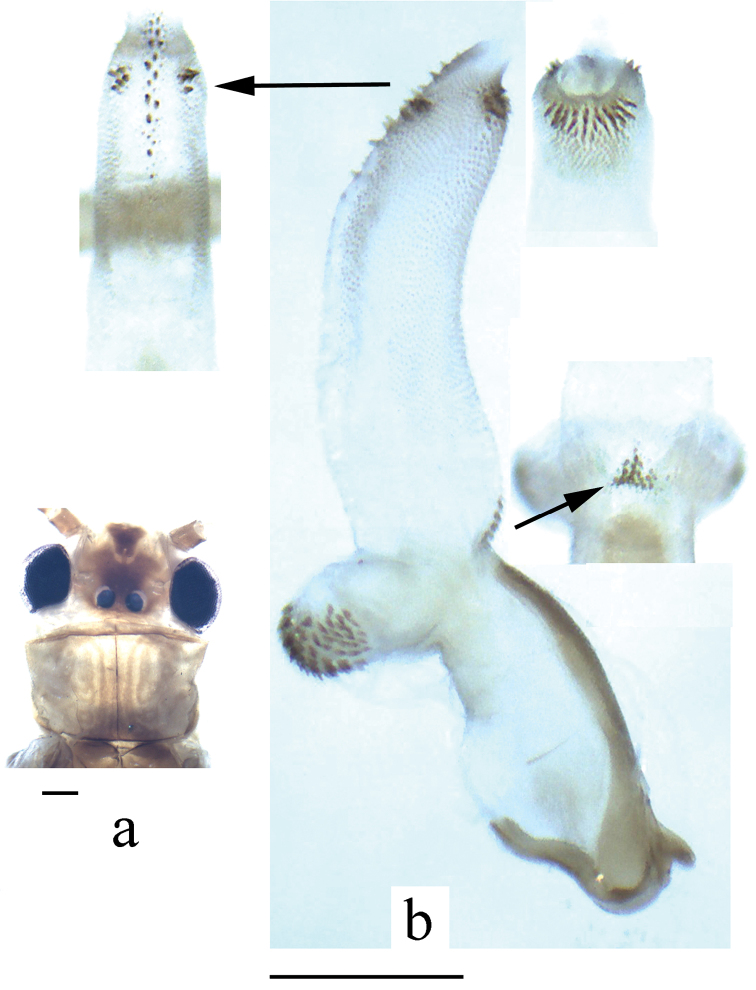
*Neoperla xuansongae*. Male **a** Head and pronotum, dorsal view **b** Aedeagus, lateral view with details in dorsal and ventral views. Scale bars: 0.5 mm.

One of the present fresh specimens of *Neoperla xuansongae* shows a heavier head pattern than types (Fig. 8a), and a small mesodorsal patch of spines at base of the aedeagal sac is also present in types but was overlooked in the original descriptions (Fig. 8c).

### Provisional key to *Neoperla* species (males) from Wuyi Moutain, Fujian Province, China

**Table:** 

1	Aedeagal tube completely sclerotized (Figs 2c, 4c & 6c)	2
–	Aedeagal tube incompletely sclerotized (Figs 3, 7 & 8)	7
2	Aedeagal tube with ventral lobes (Figs 1 & 6c)	3
–	Aedeagal tube without ventral lobes (Fig. 4c)	5
3	Aedeagal tube with slender and finger like ventral lobes	4
–	Aedeagal tube with nipple like and stout lobes (Fig. 1)	*Neoperla brevistyla* sp. n.
4	Aedeagal sac a short cone with blunt tip, head without distinct pattern (figs 25–13 in Du 1999)	*Neoperla duratubulata*
–	Aedeagal sac with a median constrict and tapering apex, head with distinct median dark brown pattern (Fig. 6)	*Neoperla qingyuanensis*
5	Posterior process of tergum 7 triangular	6
–	Posterior process of tergum 7 quadrate	*Neoperla similidella*
6	Aedeagal sac shorter than tube, covered with spinules (figs 25–12 in Du 1999)	*Neoperla breviscrotata*
–	Aedeagal sac longer than tube, covered with many large spines (Figs 4–5, figs 25–16 in Du 1999)	*Neoperla lii*
7	Aedeagal tube with lobes	8
–	Aedeagal tube without lobes (Fig. 7)	*Neoperla tuberculata*
8	Aedeagal tube with an apical lobe (fig. 14 in Li et al. 2012)	*Neoperla similiserecta*
–	Aedeagal tube without an apical lobe	9
9	Aedeagal sac with three apical patches of ventral spines (figs 8–9 in Li et al. 2013; Fig. 8)	*Neoperla xuansongae*
–	Aedeagal sac with only one apical patches of ventral spines (Fig. 3)	*Neoperla henana*

## Concluding remarks

This study was at the scale of the Wuyi Moutain Nature Reserve. Du (1999) summarized the stonefly fauna in Fujian Province and described three new *Neoperla* from the Reserve: *Neoperla breviscrotata*, *Neoperla duratubulata* and *Neoperla lii*. In the present study, an additional new species is described and added to stonefly fauna of this area: *Neoperla brevistyla* sp. n.The first records of *Neoperla henana* Li, Wu & Zhang, 2011, *Neoperla similiserecta* Wang & Li, 2012, *Neoperla qingyuanensis* Yang & Yang, 1995, *Neoperla xuansongae* Li & Li, 2013 and *Neoperla tuberculata* Wu, 1938 are given for the Reserve. The present study showed that *Neoperla* is the most species rich genus with 10 species currently known from the reserve, 6 species added after the work of Du (1999).

## Supplementary Material

XML Treatment for Neoperla breviscrotata

XML Treatment for Neoperla brevistyla

XML Treatment for Neoperla duratubulata

XML Treatment for Neoperla henana

XML Treatment for Neoperla lii

XML Treatment for Neoperla qingyuanensis

XML Treatment for Neoperla tuberculata

XML Treatment for Neoperla similidella

XML Treatment for Neoperla similiserecta

XML Treatment for Neoperla xuansongae

## References

[B1] Cao TKT Ham SA Bae YJ (2007) Description of three new species of *Neoperla* (Plecoptera: Perlidae) and a historical review of tropical Southeast Asian Perlidae. Zootaxa 1453: 41-54.

[B2] Chu Y-T (1929) Descriptions of four new species and one new genus of stone-flies in the family Perlidae from Hangchow. The China Journal 10: 88-92.

[B3] DeWalt RE Neu-Becker U Stueber G (2013) Plecoptera Species File Online. Version 5.0/5.0. 6/30/2013. http://Plecoptera.SpeciesFile.org

[B4] Du Y-Z (1999) Plecoptera. In: Huang BK (Ed) Fauna of Insects in Fujian Province of China. Vol. 3 Fujian Science and Technology Publishing house, Fuzhou, Fujian, China, 301-335.

[B5] Du Y-Z (2000a) *Neoperla magisterchoui*, a new species of the genus *Neoperla* Needham (Plecoptera: Perlidae) from China. In: Zhang YL (Ed) Systematic and faunistic research on Chinese insects. Proceedings of the 5th National Congress of Insect Taxonomy. China Agriculture Press, Beijing, 1-3.

[B6] Du Y-Z (2000b) Two new species of the genus *Neoperla* Needham (Plecoptera: Perlidae: Perlinae) from Guizhou, China. Entomotaxonomia 22: 1-5.

[B7] Du Y-Z Sivec I (2004) Plecoptera: Perlidae, Nemouridae, Leuctridae. In: Yang XK (Ed) Insects from Mt. Shiwandashan area of Guangxi. China forestry Publishing House, Beijing, 39-45.

[B8] Du Y-Z Sivec I (2005) Plecoptera. In: Yang XK (Ed) Insect Fauna of Middle-west Qinling Range and South Mountains of Gansu Province. Science Press, Beijing, 38-54.

[B9] Du YZ Sivec I He J-H (1999) A checklist of the Chinese species of the family Perlidae (Plecoptera: Perloidea). Acta Entomologica Slovenica 7: 59-67.

[B10] Du Y-Z Sivec I Zhao M-S (2001) Plecoptera. In: Wu H Pan CW (Eds) Insects of Tianmushan National Nature Reserve. Science Press, Beijing, 69-80.

[B11] Du Y-Z Wang Z-J (2005) Plecoptera: Leuctridae, Nemouridae, Perlidae and Peltoperlidae. In: Yang MF Jin DC (Eds) Insects from Dashahe Nature Reserve of Guizhou. Guizhou People Press, Guiyang, Guizhou, 51-57.

[B12] Du Y-Z Wang Z-J (2007) Nemouridae and Perlidae. In: Li ZZ Yang MF Jin DC (Eds) Insects from Mountain Leigongshan Landscape of Guizhou. Guizhou Science and Technology Publishing house, Guiyang, Guizhou, 84-90.

[B13] Li W-H Li X-P (2013) A new species of the genus *Neoperla* (Plecoptera: Perlidae) from China. Acta Zootaxonomica Sinica 38: 75-77.

[B14] Li W-H Liang H-Y Li W-L (2013a) Review of *Neoperla* (Plecoptera: Perlidae) from Zhejiang Province, China. Zootaxa 3652(3): 353-369. doi: 10.11646/zootaxa.3652.3.4 26269838

[B15] Li W-H Wang R-F (2011) A new species of *Neoperla* (Plecoptera: Perlidae) from China. Entomological News 122: 261-264. doi: 10.3157/021.122.0308

[B16] Li W-H Wang H-L Lu W-Y (2011) Species of the genus *Neoperla* (Plecoptera: Perlidae) from Henan, China. Zootaxa 2735: 57-63.

[B17] Li W-H Wang G-Q Lu W-Y (2012) Species of *Neoperla* (Plecoptera: Perlidae) from Hubei, China. Zootaxa 3478: 32-37.

[B18] Li W-H Wang G-Q Li W-L Murányi D (2012) Review of *Neoperla* (Plecoptera: Perlidae) from Guangdong Province of China. Zootaxa 3597: 15-24.

[B19] Li W-H Wang G-Q Qin X-F (2013b) Two new species of *Neoperla* (Plecoptera: Perlidae) from China. ZooKeys 290: 21-30. doi: 10.3897/zookeys.290.4568

[B20] Li W-H Wu L-M Zhang H-R (2011) A new species of the genus *Neoperla* (Plecoptera: Perlidae) from Henan, China. Acta Zootaxonomica Sinica 36: 33-35.

[B21] Sivec I Zwick P (1987) Some *Neoperla* (Plecoptera) from Taiwan. Beiträge zur Entomologie 37: 391-405.

[B22] Sivec I Stark BP Uchida S (1988) Synopsis of the world genera of Perlinae (Plecoptera: Perlidae). Scopolia 16: 1-66.

[B23] Stark BP Sivec I (2008) New species and records of *Neoperla* (Plecoptera: Perlidae) from Vietnam. Illiesia 4: 19-54.

[B24] Wu C-F (1935) Aquatic insects of China. Article XXI. New species of stoneflies from East and South China. (Order Plecoptera). Peking Natural History Bulletin 9: 227-243.

[B25] Wu C-F (1938) Plecopterorum sinensium: A monograph of stoneflies of China (Order Plecoptera). Yenching University, 225 pp.

[B26] Wu C-F (1948) Fourth supplement to the stoneflies of China (Order Plecoptera). Peking Natural History Bulletin 17: 75-82.

[B27] Wu C-F (1962) Results of the Zoologico-Botanical expedition to Southwest China, 1955–1957 (Plecoptera). Acta Entomologica Sinica 11 (Supplement): 139–153.

[B28] Wu C-F (1973) New species of Chinese stoneflies (Order Plecoptera). Acta Entomologica Sinica 16: 97-118.

[B29] Wu C-F Claassen PW (1934) Aquatic insects of China. Article XXI. New species of Chinese stoneflies. (Order Plecoptera). Peking Natural History Bulletin 9: 111-129.

[B30] Wuyi Mountain National Nature Reserve http://www.wbr.cn/

[B31] Yang C-K Yang D (1990) New and little-known species of Plecoptera from Guizhou Province (I). Guizhou Science 8: 1-4.

[B32] Yang C-K Yang D (1991) New and little-known species of Plecoptera from Guizhou Province (II). Guizhou Science 9: 48-50.

[B33] Yang D Yang C-K (1992) Plecoptera: Perlidae. In: Huang FS (Ed) Insects of Wuling Mountains area, Southwestern China. Science Press, Beijing, 62-64.

[B34] Yang D Yang C-K (1993) New and little‐known species of Plecoptera from Guizhou Province (III). Entomotaxonomia 15: 235-238.

[B35] Yang D Yang C-K (1995a) Three new species of Plecoptera from Hainan Province. Acta Agriculture Universitatis Pekinensis 21: 223-225.

[B36] Yang, D Yang C-K (1995b) Plecoptera: Perlidae. In: Wu H (Ed) Insects of Baishanzu Mountain, Eastern China. China Forestry Publishing House, Beijing, 59-60.

[B37] Yang D Yang C-K (1996) Four new species of Plecoptera from Nei Mongol. Journal of China Agricultural University 1: 115-118.

[B38] Yang D Yang C-K (1998) Plecoptera: Styloperlidae, Perlidae and Leuctridae. In: Wu H (Ed) Insects of Longwangshan. China Forestry Publishing House, Beijing, 40-46.

[B39] Zwick P (1983) The *Neoperla* of Sumatra and Java (Indonesia) (Plecoptera: Perlidae). Spixiana 6: 167-204.

